# Fostering plant protection against certain bacterial diseases through quorum-sensing signal molecules: a critical review

**DOI:** 10.3389/fpls.2025.1602573

**Published:** 2025-12-02

**Authors:** Muhammad Anwar, Annadurai Vinothkanna, Ai-Qun Jia

**Affiliations:** 1 School of Tropical Agriculture and Forestry (School of Agriculture and Rural Affairs, School of Rural Revitalization), Hainan University, Haikou, China; 2 School of Chemistry and Chemical Engineering, Hainan University, Haikou, China and Key Laboratory of Ministry of Education for Advanced Materials in Tropical Island Resources, Hainan University, Haikou, China; 3 Hainan Affiliated Hospital of Hainan Medical University, Hainan General Hospital, Haikou, China

**Keywords:** plant bacterial pathogens, virulence, QS systems, CRISPR/Cas technology, QS-CRISPR interplay, QS inhibitors, holistic plant health.

## Abstract

Quorum sensing (QS) and clustered regularly interspaced short palindromic repeats (CRISPR) systems are envisaged as revolutionary in abating plant bacterial pathogens. Bacterial cell–cell communication and plant pathogen QSSMs (quorum sensing signaling molecules) are dissected for underlying mechanisms in prominent pathogens, viz., *Pseudomonas syringae*, *Erwinia amylovora, a*nd *Xanthomonas campestris*. Biofilm formation and virulence mechanisms are critically addressed to repurpose potential QS inhibition strategies. CRISPR technologies are combined with CRISPR engineering to produce enhanced disease-resistant varieties, with potential applications. QS-CRISPR interplay for deciphering the key interactive changes in plant health management is prioritized for deliberate future research outcomes. Sustainable agricultural practices are envisaged for successful lab-to-field authentic field trials and large-scale applicability across the globe. Potential technical limitations, the need for stringent agricultural laws, and future innovations are addressed. Moreover, the cost-effectiveness, enhanced crop production, yield, and productivity hindering the above key plant bacterial pathogens are comprehensively addressed against these plant bacterial pathogens. Furthermore, a future outlook characterized by extensive outreach and global implications is substantiated regardless of regional specificity, climate change, and global warming. A decade of research on advancements in adequate plant protection is revisited to incorporate augmented approaches, including artificial intelligence (AI) and machine learning, in sustainable agriculture. The significance of the present review is based on addressing QSSMs and plant protection strategies encompassing modern molecular biological techniques.

## 1 Introduction

Quorum-sensing (QS) systems and plant bacterial pathogenesis have been recognized as a potential phenomenon in virulence, biofilm formation, and plant–pathogen interactions, which can aggravate plant health. Plant–pathogenic bacteria are characterized by possessing several QS signals comprising acyl-homoserine lactones (AHLs), diffusible signal factors (DSFs), and the second messenger cyclic di-guanosine monophosphate (cyclic di-GMP). Furthermore, the involvement of Rpf (regulation of pathogenicity factor) hybridized to cyclic di-GMP QS signaling in *Xanthomonas* spp. for escalated virulence and pathogenicity (Sundin et al., 2016). Frequent occurrences of the AHL-based QSSM, 3-oxo-C6-HSL, and C6-HSL have been documented in the majority of plant–pathogenic bacteria. However, DSF (*cis*-11-methyl-dodecenoic acid), BDSF (*cis*-2-dodecenoic acid), and CDSF (*cis*, *cis*-11-methyldodeca-2,5-dienoic acid) are reported as non-AHL molecules in plant pathogenesis (Helman and Chernin, 2015). Recently, bacterial phytopathogens involved in plant interactions have been targeted for autoinducer 1 QS inhibition (QSI) to arrest AHL production (Majdura et al., 2023). Biofilm formation and QS mechanisms for virulence in vascular plant bacterial pathogenesis are confronted by various regulatory mechanisms (Mina et al., 2019). Gene expression patterns (stress, motility, fimbriae, sulfur and tryptophan metabolism, etc.) and the type III secretion system (T3SS) involving effectors, exopolysaccharides (EPSs), and exoenzymes can be attributed to LuxI/LuxR homologues in most phytopathogenic bacteria (Mina et al., 2019; Khokhani et al., 2017; Perrier et al., 2018). Furthermore, bacterial phytopathogens are increasingly documented for disease management targeting QS systems (Sundin et al., 2016). Moreover, plant–pathogen holobiont interactions are addressed to gain deeper insights into plant immunity, and super networks generate the complexity of phytohormone-mediated immune signaling pathways (Nobori et al., 2018; Simas et al., 2025). Master signaling and inter-kingdom regulation have been reported for the involvement of LuxI/LuxR homologues such as ExpR1/ExpR2 and LasI/LasR QS systems, revealing evolutionary patterns in bacterial plant pathogens (Joshi et al., 2021). However, intrinsic and specific mechanisms and prevalence of QS systems corresponding to ubiquitous phytopathogens comprising *Pseudomonas syringae, Erwinia amylovora*, and *Xanthomonas campestris* have not been reported earlier concerning effective eradication management in crop plants. Thus, the present review aims to establish the basis of QS systems, QSSMs, CRISPR technology, and their subsequent interplay, emphasizing potential QS inhibitors and plant health. Moreover, plant health management and sustainable agriculture are congregated for the “plant health” perspective initiative by the Food and Agricultural Organization (FAO) (https://www.fao.org/plant-health-day/en).

**Figure 1:**
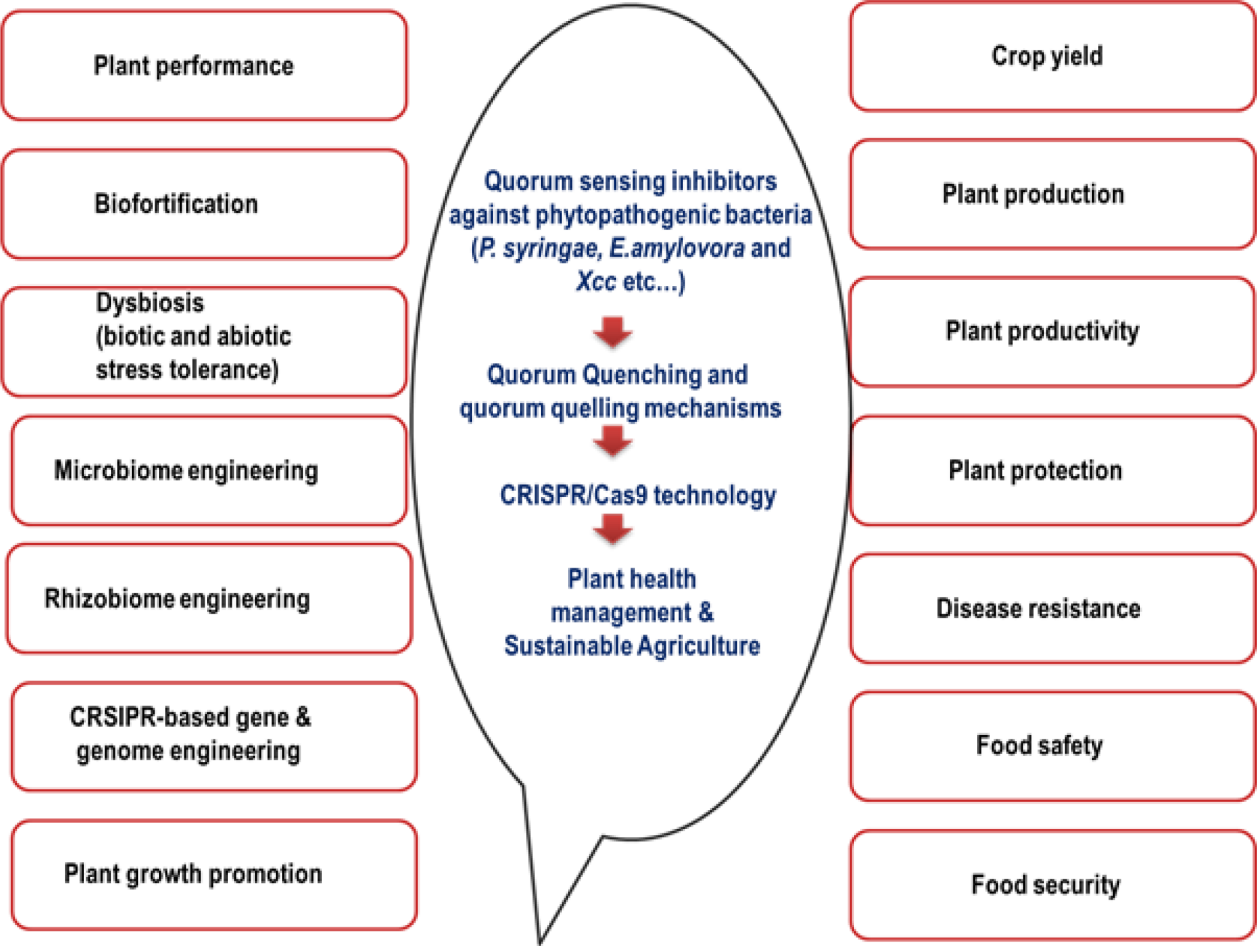
Integrated QS systems in *Pseudomonas syringae*, *Erwinia amylovora*, and *Xanthomonas campestris* and inherent future outlook for plant health management and sustainable agriculture.

CRISPR/Cas (clustered regularly interspaced short palindromic repeats –CRISPR-associated system) and plant bacterial pathogens have been harnessed in plant biology recently. Increased utilization for enhanced disease resistance against phytopathogens and targeted genome editing effectuates plant–pathogen interactions for optimal plant health (Ijaz et al., 2023). The production of disease-resistant crop varieties, encompassing rice, wheat, cucumber, cassava, cacao, grapes, citrus, apples, and bananas, has facilitated the rational development of disease resistance, surpassing conventional genetic engineering methods (Talakayala et al., 2022). The CRISPR/Cas mechanism involves the Cas endonuclease gene and short guide RNAs (sgRNAs) in the genome of the host plant for evolving targeted disease resistance to particular pathogen-resistant plants (Gosavi et al., 2020). Nevertheless, recent insights into the application of *E. amylovora* disease-resistant plants and phage resistance mechanisms require further studies to affirm the defense mechanism (Parcey et al., 2022). CRISPR technology has also been utilized for the precision diagnosis of bacterial diseases and plant genome engineering for plant disease management (Karmakar et al., 2022). Gene editing employing the CRISPR/Cas system has been ascertained for effective *X. campestris* disease resistance by knocking out susceptibility (S) genes in plant defense (Tripathi et al., 2022). CRISPR/CRISPR-associated protein 9 (Cas9) technology has been proven more effective than other methods involved in site-specific engineering [meganucleases, zinc finger nucleases (ZFNs), and transcription activator-like effector nucleases (TALENs)] (Borrelli et al., 2018). The major limitations of the above technique can be attributed to reduced sensitivity and specificity. Hence, modern molecular biology techniques are emphasized for abridging the limitations. Metabolic engineering and microbiome engineering can be foreseen as versatile applications of the CRISPR/Cas system in sustainable agriculture (Cho et al., 2018). Precise diagnosis of bacterial plant infections has also been proven effective, enabled by nano/biosensors (Zhang et al., 2022) (**Figure 1**). Consequently, interdisciplinary plant biology—encompassing synthetic biology, multiplex editing, and directed evolution—is employed to develop plants resistant to phytopathogens (Zhu et al., 2020). Moreover, disease resilience crops with enhanced agronomic traits will enable the technology to derive disease-resistant and resilient crops, mitigating both biotic and abiotic stresses. Consequently, interdisciplinary plant biology—encompassing synthetic biology, multiplex editing, and directed evolution—is employed to develop plants resistant to phytopathogens (Yadav et al., 2022). Thus, the significance of CRISPR/Cas systems can be foreseen for explicit management of sustainable crops with disease resistance and improved properties, ensuring food security and safety. Furthermore, this review compiles the critical interplay mechanisms between QS-CRISPR systems in the efficient management of plant bacterial pathogens. Pathogenesis and virulence of plant pathogenic bacteria have been studied mainly for QS mechanisms and associated complex interplay (Ansari and Ahmad, 2018). Therefore, QS inhibitors targeting plant bacterial pathogens will facilitate a comprehensive plant health management approach that emphasizes “plant health” (https://www.fao.org/plant-health-day/en) and, consequently, the overall health of the planet. As a “*priority*”, QS systems in ubiquitous plant pathogens *P. syringae*, *E. amylovora*, and *X. campestris* are critically compiled for future outlook and effective management (**Figure 1**).

**Figure 2:**
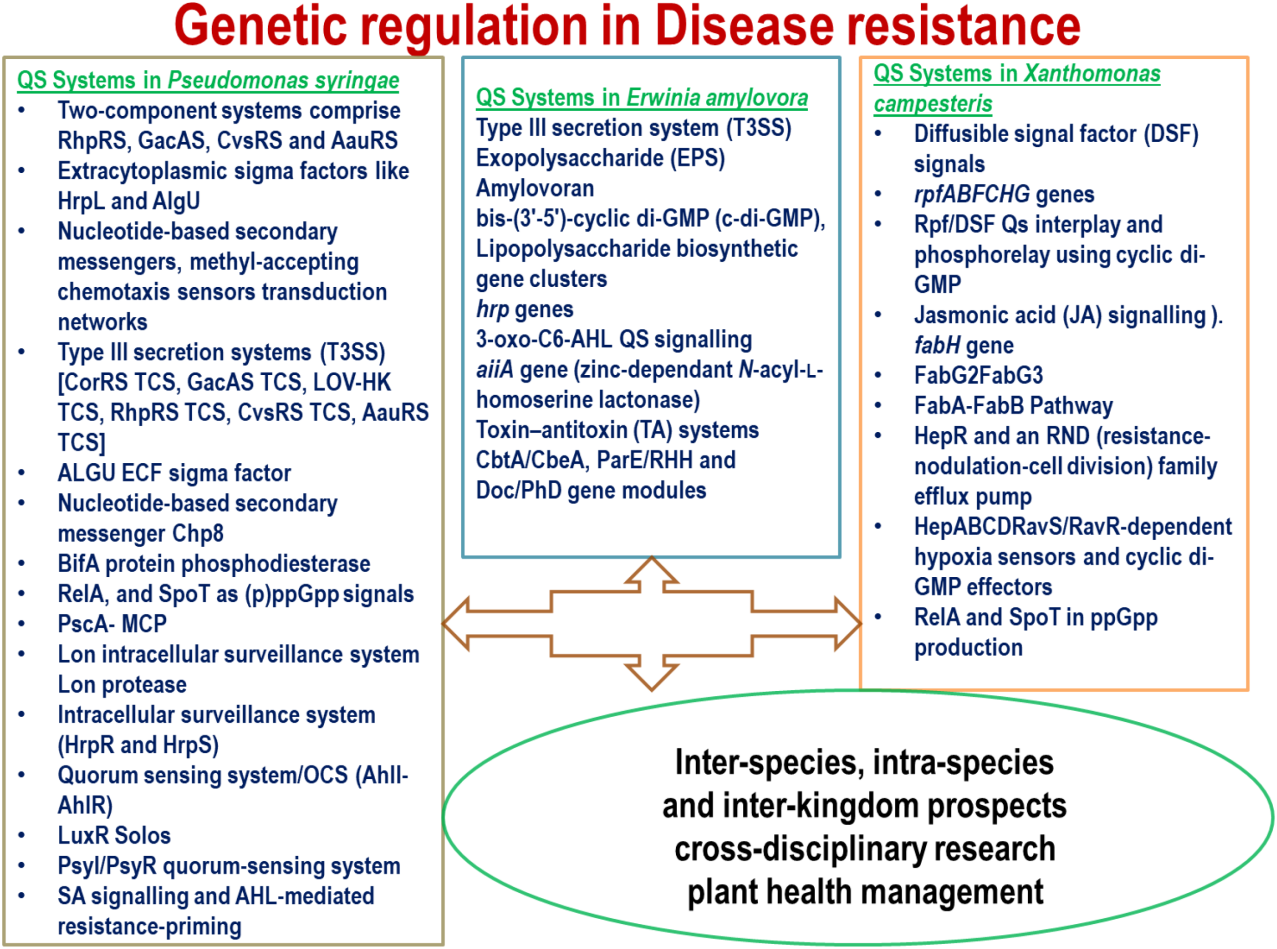
Genetic regulation of quorum-sensing systems in *Pseudomonas syringae*, *Erwinia amylovora*, and *Xanthomonas campestris*, fostering key genes for inter-species, intra-species, and inter-kingdom perspectives managing plant health.

The significance of the pathogenesis profiles of the above pathogens reveals complex and intricate virulence mechanisms. Briefly, the bacterial pathogens chosen (*P. syringae*, *E. amylovora*, and *X. campestris*) are enumerated for significant molecular plant pathology. *P. syringae* represents various strains infecting a wide range of plant hosts from grass to arboreal plants (Baltrus et al., 2017). Pathogenic profiles include *hrp/hrc* gene clusters, syringomycin, syringolin, and mangotoxin clusters encoded by the T3SS system. The signal cross-talk has been elaborated for a vast range of host and bacterial cankers (Ruinelli et al., 2019). The strains belonging to the viable but not culturable (VBNC) category of *P. syringae* comprise several pathogenesis-associated factors. The inclusive mechanisms include ACE (acetosyringone); POX (peroxidase); MCP (methyl-accepting chemotaxis protein); NADH (nicotinamide adenine dinucleotide hydrite); ABC (ATP-binding cassette transporter system); T3SS; RND [resistance/nodulation/division family representing multidrug resistance (MDR) efflux pumps]; and MarR, LysR, and Lrp/AsnC transcription factors (Postnikova et al., 2015). *E. amylovora* infections are characterized by the T3SS system, EPS amylovoran, levan, cyclic di-GMP (c-di-GMP), and small, noncoding RNAs (sRNAs). Furthermore, Hfq-dependent sRNA OmrAB/Hrs6 (negative) and ArcZ/RprA (positive) are involved in regulating the ams operon (Kharadi et al., 2021). Moreover, LPS biosynthetic gene clusters are also involved in the pathogenicity of *E. amylovora* (Piqué et al., 2015). The key pathogenic components in *X. campestris* include cellulase, mannanase, pectinase, protease (T2SS), effector proteins, and EPSs (Liao et al., 2019). The notable T2SS (*xps* and *xcs*) and *xps* genes are associated with pathogenicity. The *hrp* gene cluster encoding a T3SS has largely corresponded to pathogenesis along with the *rpf*, *gum*, and *wxc* genes regulating xanthan gum and lipopolysaccharides in *X. campestris* (Vicente and Holub, 2013) (**Figure 2**). Thus, the pathogenesis profiles of the bacterial pathogens employed in the review are summarily discussed.

## 2 QS systems in *P. syringae*

**Figure 3:**
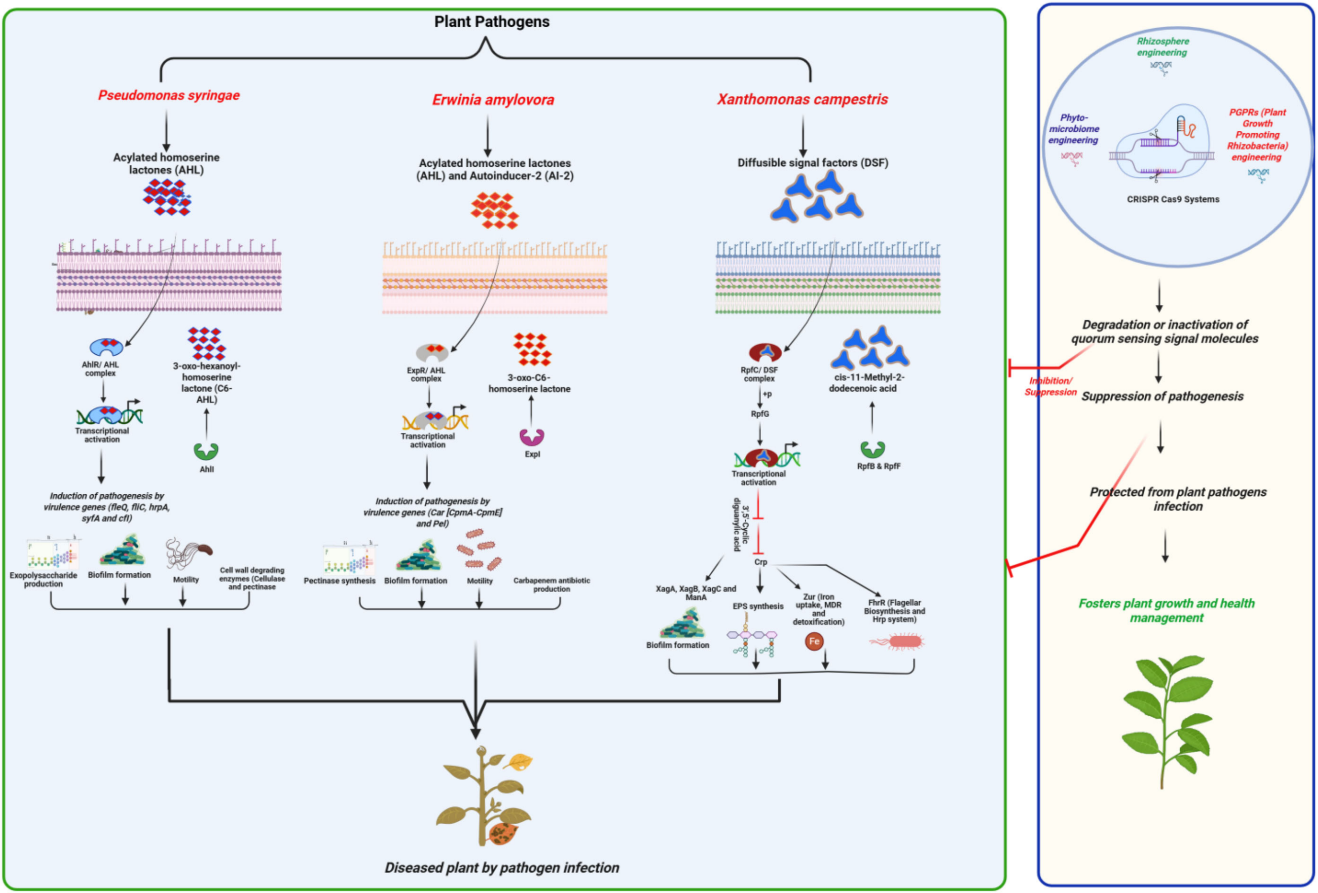
Quorum-sensing signal molecules mediated disease manifestation in the phytopathogens (*Pseudomonas syringae*, *Erwinia amylovora*, and *Xanthomonas campestris*) and CRISPR/Cas technology for plant health augmentation.

The phytopathogen *P. syringae* accounts for a complex and meticulous signal network cascade. The inherent two-component systems comprise RhpRS, GacAS, CvsRS, and AauRS. Furthermore, extracytoplasmic sigma factors, such as HrpL and AlgU, together with nucleotide-based secondary messengers and methyl-accepting chemotaxis sensors, are assembled into transduction networks (Xie et al., 2020). The potential pathovars are ubiquitous and cause enormous economic loss and severe threats to food safety and security (Xin et al., 2018). Moreover, the pathogen employs T3SS. CorRS TCS correlates to temperature changes involving CorR, which activates COR biosynthesis and *hrpL* expression (Peñaloza-Vázquez and Bender, 1998). GacAS TCS encompasses the accurate regulation of GacA concerning virulence-associated pathways, including AHL synthesis, T3SS, and swarming motility (O’Malley et al., 2020) (**Figure 2**). LOV-HK TCS is composed of blue light as a secondary messenger, reducing transcription of several alternative sigma factor genes (rpoN, rpoS, and rpoD), T3SS genes (hrpE, hopAA1-1, hrpL, and hopL1), and modulating swarming motility (Wang et al., 2013). RhpRS TCS involves the phosphorylation of RhpR, which suppresses the expression of *hrpRS* and *lon*. RhpR regulates twitching motility, c-di-GMP level, swimming motility, lipopolysaccharide production, and biofilm formation. Furthermore, phosphorylation alters RhpR regulation by alcohol dehydrogenase activity, anthranilate synthase activity, cytochrome *c*550 accumulation, and protease production (Xie et al., 2019; Deng et al., 2014; Zhou et al., 2016). CvsRS TCS comprises Ca^2+^ as a secondary messenger affecting bacterial virulence and metabolism (T3SS, alginate production, cell attachment, swimming, and swarming motility) (Fishman and Filiatrault, 2019; Anderson et al., 2014) (**Figures 2** and **3**). AauRS TCS corresponds to acidic amino acid activation of transcription of *hrpRS* and promotion of bacterial virulence in *Arabidopsis* (Yan et al., 2020). HrpL belongs to the ECF sigma factor, activating the expression of most T3SS genes. Nevertheless, HrpL accounts for the spontaneous negative regulation of gene expression (Waite et al., 2017). AlgU ECF sigma factor depends on external osmotic pressure for alginate production, flagella biosynthesis, T3SS, type VI secretion, and oxidative stress responses (Bao et al., 2020; Markel et al., 2016). The iron starvation ECF sigma factor enables secondary messengers, such as iron ions, to control the regulation and secretion of PSPTO_1203 and the uptake of pyoverdine AcsS (Psyr_2580), as well as the regulation and secretion of achromobactin (Greenwald et al., 2012). The nucleotide-based secondary messenger Chp8 utilizes c-di-GMP acting as a diguanylate cyclase to synthesize c-di-GMP. Furthermore, inhibition of flagellin production upregulates EPS synthesis. Thus, synthesized c-di-GMP empowers T3SS, flagellar assembly, EPS synthesis, siderophore biosynthesis, and oxidative stress resistance (Aragón et al., 2015). Similarly, BifA corresponds to the nucleotide-based secondary messenger c-di-GMP, contributing to BifA protein phosphodiesterase in c-di- GMP degradation, *in vivo* (Aragón et al., 2015). The RelA and SpoT act as (p)ppGpp signals using GTP and ATP. SpoT belongs to the bifunctional protein for the synthesis and hydrolysis of (p)ppGpp. Moreover, (p)ppGpp regulation involves multiple processes for virulence and survival (nucleotide/amino acid/fatty acid metabolism, EPSs production, type VI secretion system, phytotoxin production, T3SS, swarming motility, pyoverdine production, stress resistance, and cell sizes) (Wang et al., 2020) (**Figure 2**). PscA- MCP uses acidic amino acids to control swarming motility, biofilm formation, c-di-GMP production, and bacterial virulence (Cerna-Vargas et al., 2019). The Lon intracellular surveillance system Lon protease degrades T3SS activator HrpR. A cluster of T3SS effectors (AvrPto, HopPtoM, and HopPsyA) acts as a T3SS repressor. The DNA-binding transcriptional regulator Lon mediates copious metabolic pathways (1-dodecanol oxidation, glucokinase activity, and pyoverdine production). The Lon protease cleaves T3SS effectors (AvrB2, HrpW1, and HrcV) in KB and degrades metabolic factors like NuoI and NoxB) in MM (Zhou et al., 2016). The intracellular surveillance system (HrpR and HrpS) results in heterodimer formation and activates the transcription of *hrpL*. HrpS accounts for the regulation of T3SS, motility, and biofilm formation. Furthermore, the modification of the HrpS protein by sulforaphane and the suppression of the T3SS system attenuate bacterial virulence (Wang et al., 2020). QS system/OCS (AhlI–AhlR) involves 3-oxo-hexanoyl-homoserine lactone. AHL signaling results in stable complex formation with AhlR and, furthermore, the activation of the expression of *ahlI*. The AhlI–AhlR system is independently dependent on AefR and GacA activation (Quiñones et al., 2004).

*P. syringae* pathovar *tabaci* 11528 (*P. syringae* 11528) proved that the AHL-mediated QS system affirms plant–microbe interactions conferring potential attributes (flagella, chemotaxis, pilus, extracellular polysaccharides, secretion systems, and two-component system). Thus, early colonization and latency of pivotal infections are ascertained (Xie et al., 2020; Cheng et al., 2017) (**Figure 2**). The heterogeneity of QS systems is confirmed for the diffusible signal *P. syringae* for leaves with implications for successful evolutionary strategies (Pérez-Velázquez et al., 2015). Comparatively, three LuxR solos have been reported for emerging infections incited by *P. syringae* pv. *actinidiae* in kiwi fruit (Patel et al., 2014). Similarly, AHL homologues have been associated with effective virulence and pathogenicity in reconfiguring the phyllosphere in kiwi fruits (Cellini et al., 2020). AHLs comprising *N*-acyl-homoserine lactones escalate oxidative burst, hypersensitive response, cell wall strengthening, and metabolites for active plant defense, accounting for innate immunity (Cheng et al., 2018). The recent consolidation of QS systems has been attributed to the gene expression of *rsmX* and *rsmY. Moreover, small non-coding RNAs, including P. syringae* aggravations, decipher canonical stop codon for *psyI* in *Pto*DC3000 for AHL similarities (Nakatsu et al., 2019) (**Figure 2**). Linalool’s interactive perspectives reveal the modulation of the PsyI/PsyR QS system in addressing the virulence of *P. syringae* pv. tomato DC 3000 (Deepa et al., 2022). Similarly, GacS/GacA TCS indicated that *rsmZ* and *rsmY* transcription was hindered by bacterial QS mechanisms (Zhang et al., 2019)*. P. syringae pv. tabaci* 11528 showed *N*-(3-oxo-hexanoyl)-homoserine lactone-based QS regulation and optimal gene expression profiling (Cheng et al., 2016). The complexity of exogenous QS signaling was enumerated by employing phage defense through OmpV expression regression in *P. syringae* pv. *actinidiae (*Ou et al., 2025*).* The SA signaling pathway was also redressed for AHL-mediated resistance priming in *P. syringae* pv. *tomato* DC3000 (*Pst*DC3000), as indicated by 3-O-C8-HSL potentiation (Liu et al., 2020) (**Table 1**; **Figures 2**, **3**). Thus, a critical assessment of *P. syringae* QS systems is summarized to elucidate the complexities of a holistic phenomenon and mechanistic signaling cascade.

**Table 1:** Quorum-sensing inhibitors (QSIs) against plant pathogens *Pseudomonas syringae, Erwinia amylovora*, and *Xanthomonas campestris*. .

S. no.	Name of the QSI	Quorum-sensing systems	Mechanism of action	References
*Pseudomonas syringae*				
1.	Phytochemicals	AHL-based QS systems	AHL degradation and disruption of QS signaling attributed to preliminary screening assessments	TruChado et al., 2015
2.	QSI effects of *Bacillus* and *Variovorax* species	*N*-tetradecanoyl homoserine lactone and *N*-hexanoyl homoserine lactone	AHL degradation causes motility inhibition, biofilm formation, and arrest of virulence factors	Jose et al., 2019
3.	Endophytic bacteria (*Bacillus cereus* Si-Ps1 and *Pseudomonas azotoformans* La-Pot3-3) mediated generalized QS inhibition	*aiiA* gene degradation	Quorum-quenching (QQ) activities against *Pseudomonas syringae* pv. *syringae* (*Pss*) B728a biofilms and AHL degradation due to the *aiiA* gene	Akbari Kiarood et al., 2020
4.	Extracellular and intracellular AHL-lactonase	AHL QS system	*Bacillus cereus* INT1c biocontrol resulting in QQ activities	Ananda et al., 2019
5.	Linalool	PsyI/PsyR QS system	Suppression of the AHL QS system in *Pseudomonas syringae* pv. Tomato DC 3000 virulence at 80 ppm and modulation	Deepa et al., 2022
6.	*Pseudomonas syringae* pv. *actinidiae* (*Psa*) bacteriophages	AHL QS system	Repression of OmpV expression, indicating non-AHL QS signals	Ou et al., 2025
7.	Hexanoic acid	Type III secretion system	*Enhanced gene expression of cfl, cfa1, hrpL, hrpA*, and *avrPtoB contributing to* virulence, survival, and pathogenicity attenuation	Scalschi et al., 2014
8.	Potassium permanganate and *P. syringae* pv. *syringae* mangotoxin	Leudiazen signals in AHL QS systems	Organic farming mediated dissection of QS signaling at the preliminary level	Sieber et al., 2021
*Erwinia amylovora*				
9.	HAMD-MOL and MeOH-MOL	AHL system	Decreased amylovoran synthesis, biofilm formation, and QS inhibition against *E. amylovora*	Fontana et al., 2022
10.	Piericidin A and glucopiericidin from *Streptomyces xanthocidicus* KPP01532	AHL system	QS inhibition of virulence genes *pelC*, *pehA*, *celV*, and *nip* in *Erwinia carotovora* subsp*. atroseptica*	Kang et al., 2016
11.	QSIs through the antagonistic activity of *Pseudomonas protegens* 59M	AHL system	*The expression of phlD, pltC, pltB*, and *pltC* involves siderophore production, IAA, HCN production, and AHL degradation	Mikiciński et al., 2024
12.	*Bacillus simplex*	AHL system	QQ activities of the *aiiA* gene encoding zinc-dependent AHL silencing in *E. amylovora* sy69	Hanano et al., 2014
14.	Antagonistic activity of *Pseudomonas orientalis* F9	LasI/R and RhlI/R QS systems	The pyoverdine, safracin, and phenazine mutations caused inhibition of *E. amylovora* in apple flowers.	Santos Kron et al., 2020
*Xanthomonas campestris*				
15.	QSI activities of *Burkholderia anthina* HN-8	DSF system	11-methyl-2-dodecylene acid degradation by *B. anthina* HN-8 and QQ activities	Ye et al., 2020
16.	Chumacin-1 and Chumacin-2	DSF system	*rpfB-dependent QS regulation*	Song et al., 2022
17.	Trans-2-decenoic acid methyl ester	DSF system	DSF degradation by *Cupriavidus* sp.	Ye et al., 2019a
18.	QSI of *Acinetobacter lactucae* QL-1	DSF system	*Xcc* virulence attenuation by QQ enzymes and plant protection against DSF-dependent pathogens	Ye et al., 2019b
19.	*Moringa oleifera* Lam leaf extracts	DSF system	QS inhibition against *Xanthomonas campestris* pv. *campestris* (Xcc) (swarming motility, and biofilm formation)	Fontana et al., 2021
20.	Thymol-loaded chitosan nanoparticles (TCNPs)	DSF system	QS inhibition through exopolysaccharides and xanthomonadin production against *Xcc*	Sreelatha et al., 2022

## 3 QS systems in *E. amylovora*

*E. amylovora* belongs to the *Enterobacteriaceae* family, constituting Gram-negative phytopathogens affecting wide plant hosts in the Rosaceae (apple and pear), resulting in global consequences to plant health. The potential virulence factors include the QS systems, which are aggravated to the T3SS, the EPS amylovoran, biofilm formation, and motility. Furthermore, complexities are associated with bis-(3′-5′)-cyclic di-GMP (c-di-GMP), lipopolysaccharide biosynthetic gene clusters, and *hrp* genes conferring virulence (Piqué et al., 2015) (**Figures 2** and **3**). Antagonistic mechanisms like siderophore, IAA, and HCN production have also been considered in *E. amylovora* infections in pear fruitlets affected by fire blight (Mikiciński et al., 2024) (**Table 1**). Specific humidity and temperature aggravate the manifestation of fire blight by *E. amylovora* (Pedroncelli and Puopolo, 2024). Thus, deeper insights are to be shed on the emerging phytopathogen for the eradication of devastating fire blight disease and ecological impacts. 3-oxo-C6-AHL QS signaling was attributed to the *aiiA* gene (zinc-dependent *N*-acyl-l-homoserine lactonase) corresponding to AHL-based QS systems and silencing mechanisms in *E. amylovora* sy69, *in vitro vitro* and *in planta* (Hanano et al., 2014) (**Figures 2**, **3**). Variable number of tandem repeat sequences (VNTRs) analysis revealed biofilm, siderophores, and biosurfactant production, motility, and environmental effects on growth in *E. amylovora* (Tafifet et al., 2020). Hence, the pathogen requires additional research based on modern molecular techniques, including CRISPR technology, to establish the intricate molecular mechanisms of pathogenicity and virulence (**Figure 3**). Furthermore, toxin–antitoxin (TA) systems in *E. amylovora* showed CbtA/CbeA, ParE/RHH, and Doc/PhD gene modules associated with plasmid stability, stress management, biofilm formation, and antibiotic persistence, revealing functional diversity and specificity (Shidore et al., 2019) (**Figure 2**). Inter-species interactions in the plant host holobiont have been stressed for *E. amylovora* and increased association of disease incidence and severity (Hassani et al., 2024). Nevertheless, *E. amylovora* was chosen in the present review for establishing the future prospective cross-disciplinary research among phytopathogens. Moreover, the inter-species, intra-species, and inter-kingdom prospects are necessitated for revitalizing the QS systems in floral phytopathogens and environmental spread in assuring plant health management. Horizontal gene transfer mechanisms can aggravate bacterial pathogenesis and complex virulence systems. Thus, QS system assessments for the pathogens are conducted for intricate mechanisms.

## 4 QS systems in *X. campestris*

DSF is the most reported QS system in *X. campestris*, followed by *rpfABFCHG* genes. Furthermore, the interplay between Rpf/DSF QS systems is mediated by phosphorelay mechanisms that are based on cyclic di-GMP levels. Moreover, sucrose and glucose levels enhance RpfB activity through the salicylic acid pathway, thereby increasing DSF signals (Zhang et al., 2019) (**Figures 2** and **3**). Thus, DSF biosynthetic enzymes, including DSF synthesis and RpfF protein based on glutamate, are associated with DSF signaling QS systems in *X. campestris* (Feng et al., 2023). The bacterial DSF belongs to a low-activity QS signal molecule that exhibits increased binding affinity to histidine kinase RpfC, thereby triggering RpfC autophosphorylation. Further breakdown of bacterial cells by RpfB suppresses the regulation of RpfC enzyme activities and homologous response regulator RpfG, encoding c-di-GMP hydrolase (Tian et al., 2022). Moreover, DSF signals are ascertained for priming plant immune responses against *X. campestris pv. campestris (Xcc)* involving jasmonic acid (JA) signaling in *Brassica oleracea* and *Arabidopsis thaliana* (Zhao et al., 2023) (**Figure 2**). *The fabH* gene was shown to be involved in DSF QS signaling, which contributes to the control of black rot disease in cruciferous vegetables caused by *Xcc* (Yu et al., 2016). Repressive regulation of the co-evolution of the DSF QS system-based plant innate immunity was evidenced by the EPS xanthan in *X. campestris* (Kakkar et al., 2015). FabG2, the fatty acid synthesis enzyme comprising 3-hydroxyacyl-acyl carrier protein (3-hydroxyacyl-ACP), conferred long-chain specificity for DSF signaling in *Xcc* (Hu et al., 2018). Furthermore, FabG3 involving 3-oxoacyl-ACP reductase was corroborated by xanthomonadin biosynthesis in *Xcc* virulence (Yu et al., 2019) (**Table 1**; **Figure 2**). Nonetheless, the FabA–FabB pathway was found to be associated with modulating DSF synthesis in *Xcc* (Yu et al., 2023). HepR and an RND (resistance-nodulation-cell division) family efflux pump HepABCD were depicted in *Xcc* involving salicylic acid efflux and sensor regulation encoding virulence (Song et al., 2024). RpoN1 and RpoN2 correspond to the homologous regulators correlating to the regulation of virulence, flagella synthesis, and basal metabolism in *Xcc*, revealing the specificity of transcription (Li et al., 2020). Apart from DSF signaling, RavS/RavR-dependent hypoxia sensors and cyclic di-GMP effectors were reported in *Xcc* (He et al., 2020). Plant–pathogen interactions in *Xcc* and host plants have been affirmed for salicylic acid activation of RpfB QS systems in *Xcc* (Song et al., 2022) (**Figure 3**). The complexity of *Xcc* virulence was also attributed to the light sensor mediated through the photoreceptor, bacteriophytochrome (Bonomi et al., 2016). The RelA and SpoT in ppGpp production were compromised to virulence, pathogenesis, stress tolerance, and growth regulation in *Xcc* (Bai et al., 2021). Hence, intricate insights for deciphering *Xcc* responses with plant holobiont interactions require further assessment. Therefore, the complex and widespread phenomenon of SA–JA cross-talk could be utilized in CRISPR-QS systems to prevent phytopathogenicity, ensuring plant health effectively. Hence, QSSMs and CRISPR systems, which enable potential scenarios for plant health, are addressed further.

## 5 QSSMs and CRISPR systems in plant health

Communication between bacteria and signaling molecules such as AHLs, AIPs, and AI-2 depicts the significance of pathogenesis. Nonetheless, the target genes for AHL, AIPs, AI-2 bacterial signal molecules, and QS systems emphasize the reality of CRISPR systems in confirming plant health (Filik and Filik, 2023) (**Figure 3**). Rice (*Oryza sativa*) remains an extensively researched crop, establishing the potent applications of CRISPR systems in augmenting plant performance, biofortification, and combating dysbiosis (biotic and abiotic stress tolerance) (Ricroch et al., 2017). Microbe–plant interactions involving microbiome and rhizobiome engineering dissect sustainable agriculture for plant protection and plant growth promotion (PGP) in the era of CRISPR tools (Shelake et al., 2019) (**Figure 1**). Disease-resistant plant engineering has been made at ease employing CRISPR-Cas9 technology, stressing pathogen-associated molecular pattern (PAMP)-triggered immunity (PTI) and/or effector-triggered immunity (ETI) (Tyagi et al., 2021). CRISPR/Cas9-targeted modification has foreseen escalated applications utilizing meganucleases, ZFNs, TALENs, and CRISPR/Cas9. Moreover, genetic manipulations against the majority of crops, including rice, tomato, wheat, and citrus, are given prominent importance (Borrelli et al., 2018). CRISPR genome editing provides evidence of both climate resilience and disease resistance, ensuring plant health. Thus, the future of food safety, security, and crop protection is primarily attributed to CRISPR technology (Zaidi et al., 2020). CRISPR/Cas genome editing technology is applied for enhanced yield production, productivity, disease resistance, herbicide resistance, plant breeding, and fast-tracked domestication (Zhu et al., 2020). The genome editing using CRISPR technology will be utilized for generating bio-editing and plant breeding in sustainable agriculture (Langner et al., 2018). Nevertheless, nanotechnology incorporations with CRISPR technology could revolutionize the development of climate change-resilient crops in the future (Demirer et al., 2021). However, ethical issues and stringent global laws are envisaged for application perspectives of CRISPR technology (Zhang et al., 2020). Moreover, global agriculture is emphasized for improved plant health and food security (Tyagi et al., 2020) (**Figures 1** and **3**). Therefore, QS-CRISPR interplay could aggravate the closing in of effective plant health management and the future of sustainable agriculture.

## 6 QS and CRISPR interplay and plant health management

Plant health management involving the exploration of plant–microbe interactions has been stressed for sustainability in agricultural practices using CRISPR technology (Shelake et al., 2019). Furthermore, the emphasis on CRISPR/Cas9 technology was adjudicated as the potential tool in the establishment of PGP, plant protection, and climate-resilient farming practices (Prabhukarthikeyan et al., 2020). The advantages of using CRISPR/Cas technology were foreseen as an explicit disease management strategy owing to escalated precision, robust technique, minimal off-targets, and focusing multiple targets (Bansal et al., 2022). Food safety, security, and plant disease management protocols are foreseen as a potential future in terms of cost-effectiveness, specificity, and sensitivity using CRISPR/Cas technology (Wagh et al., 2021). Engineering microbiomes for large-scale applications with disease resistance has been emphasized by employing high-throughput gene editing technology, augmenting disease resistance. Hence, the futuristic implications of CRISPR/Cas9 technology rely on plant defense engineering to ensure global food safety (Tyagi et al., 2021). Nevertheless, a complex interplay of phytopathogens involving host plants, bacteria, and viruses is stressed in sustainable agriculture. Hence, soil health, biogeochemical cycling, food security, and transgenesis using CRISPR technology pose pivotal significance (Astapati and Nath, 2023). Preharvest and postharvest management of agricultural losses and economic food security at global levels are enriched for environmental-friendliness concerning plant–pathogen interactions (Ezrari et al., 2024).

A holistic mechanism of CRISPR technology involves plant protection, abiotic stress management (including drought, salinity, and heat), and PGP. Hence, empirical systems biology in the elucidation of microbial, genetic, and metabolic interactions comprises signaling pathways underlying plant–microbe interactions (Manzar et al., 2022). Food loss and the circular economy approach rely upon CRISPR technology. Thus, a multifaceted gene editing strategy can facilitate water valorization and food and feed production, and minimize greenhouse gas (GHG) emissions (Hemalatha et al., 2023). CRISPR/Cas9 technology gene editing has been witnessed as an effective horizon for crop improvement and plant stress mitigation (Patra et al., 2024). RNAi-induced double-stranded RNA (dsRNA) technology reveals potent development of biopesticides and disease-resistant plants (Halder et al., 2022). The increasing global demand for food and the escalating levels of crop production and productivity necessitate the utilization of CRISPR genome engineering technology (Ali et al., 2022). Furthermore, abiotic stress management relies highly on the CRISPR/Cas9 gene editing strategy (Kumar et al., 2023). An update on CRISPR/Cas9 gene editing in tomatoes outlined dysbiosis management involving abiotic and biotic stresses (Chandrasekaran et al., 2021). The CRISPR strategy could enable nano-biofertilizers/nano-pesticides to decipher interplay with plant-associated microbiomes (Ahmed et al., 2023). Hence, multi-omics approaches and CRISPR technology can facilitate the deciphering of rhizobiomes contributing to plant health and resilient agricultural practices (Dukare et al., 2022). However, challenges and limitations require a broad-spectrum viewpoint in evaluating the effectiveness of QS systems and CRISPR cross-talk in mitigating the spread of phytopathogenic bacteria (**Figure 3**).

CRISPR technology, harnessing QS, can be utilized to interfere with plant pathogens at the inter-kingdom level (Joshi et al., 2021). Furthermore, biotic stresses can be hindered by biocontrol agents engineered for determining plant protection (Ayaz et al., 2023). Broad host-range (BHR) plasmids were proposed for genome engineering in affirming plant–microbiome interactions using CRISPR systems for efficient plant growth (Ke et al., 2021). The CRISPR/Cas system has been utilized for QS-based disease control and plant health management in *Xanthomonas citri* (Martins et al., 2024) and *E. amylovora* involving phage biocontrol (Parcey et al., 2022). Novel plant varieties can be developed using rhizosphere engineering of QS molecules (Li et al., 2023) and phyto-microbiome engineering utilizing CRISPR technology (Patyal et al., 2025). CRISPR interference can be used in climate-resilient agricultural engineering for generating plant holobionts and novel sustenance (Portal-Gonzalez et al., 2025). Similarly, CRISPR technology was affirmed for PGPR (plant growth-promoting rhizobacteria) engineering and plant interactions in Green Revolution 2.0 (Singh and Ramakrishna, 2021) (**Figure 3**). Thus, CRISPR technology in plant–microbe interactions concerning QS systems will pose a novel thrust in the future.

## 7 QS inhibitors against plant bacterial pathogens

QS I has gained attraction in the effective arrest of phytopathogenic bacteria. Natural product research and quorum quenching (QQ) mechanisms largely rely on elimination in lab- and field-scale approaches. Plant extracts and phytochemicals disrupt bacterial pathogenesis and down-regulate QSSMs (TruChado et al., 2015). Auto-inducers like *N*-acyl-homoserine lactones (AHLs) are emphasized in inter-kingdom QS signaling. Hence, QQ and QSI, which target LuxR solo genes, are utilized by plant growth-promoting bacteria (PGPB) for potential biocontrol (Hartmann et al., 2021) (**Figure 3**). QS inhibitors are stressed as anti-pathogens, enabling wide-scale field trials in treating plant infections (Kalia et al., 2019). Natural compounds inhibiting QS systems are addressed to biofilms, hydrolytic enzymes, toxins, and plasmids contributing to virulence (Gutierrez-Pacheco et al., 2019). Pre-harvest and post-harvest microbial control is often signified by natural compounds preventing spoilage and preservation through QSI (MaChado et al., 2020). Phytopathogens utilize AHLs, DSF, and 3-OH-PAME/3-OH-MAME molecules for QS regulation, establishing virulence and subsequent QQ mechanisms for plant protection (Baltenneck et al., 2021). Moreover, QSI has also been highlighted in Mediterranean plant essential oils belonging to the Lamiaceae and Verbanaceae families, which combat phytopathogens (Camele et al., 2019). QS interference against *P. syringae* pv. *passiflorae* was affirmed *by Bacillus* and *Variovorax* species degrading AHLs, conferring plant protection (Jose et al., 2019). Endophytic bacteria (*Bacillus cereus* Si-Ps1 and *Pseudomonas azotoformans* La-Pot3-3) *Citrus sinensis* and *C. sinensis* var. Thomson’s leaf cultivars revealed QQ activities against *P. syringae* pv. *syringae* (*Pss*) B728a. The corresponding mechanisms were attributed to reduced biofilms and AHL degradation as evident from the presence of *the aiiA* gene (Akbari Kiarood et al., 2020). Similarly, *P. syringae* was inhibited by QQ mechanisms containing AHL-lactonase from *B. cereus* INT1c, depicting competitive inhibition (Ananda et al., 2019). Linalool suppressed the AHL QS system in *P. syringae* pv. Tomato DC 3000, which contributed to reduced virulence at 80 parts per million (ppm). Moreover, the computational assessment showed the modulation of the PsyI/PsyR QS system (Deepa et al., 2022). *P. syringae* pv. *actinidiae* (*Psa*) bacteriophages were effectively proven for repression of OmpV expression showing non-AHL QS signal involvement (Ou et al., 2025) (**Table 1**; **Figure 3**). *P. syringae* pathovars’ anti-QS mechanisms are summarized for symbiosis, pathogenicity, competence, conjugation, antibiotics, motility, sporulation, and biofilm inhibition (Manikandan et al., 2023). Hexanoic acid acts as an inducer of resistance in attenuating *P. syringae* pv. tomato DC3000 virulence, survival, and pathogenicity (Scalschi et al., 2014). *P. syringae* pv. *syringae* mangotoxin signaling molecules were mitigated using a combinatorial strategy employing potassium permanganate and organic farming, degrading the leudiazen signals (Sieber et al., 2021). *3-O-C6-HSL QSSM was fostered for AHL degradation in P. syringae pv. tabaci* 11528 (Cheng et al., 2016). The endophytic fungus *Alternaria leptinellae* E138 was depicted for *P. syringae* QSI in tomato (García-Latorre et al., 2024). *Thymus vulgaris* (thyme)- and *Origanum vulgare* (oregano)-derived essential oils inhibited *P. syringae* and showed potential QSI (biofilm formation, coronatine, syringomycin, and tabtoxin production).Furthermore, it was designed to inhibit TA systems and prevent virulence (Carezzano et al., 2017). *Pseudomonas aeruginosa* PAO1 was inhibited by *Artemisia argyi* leaf extracts, posing QSI of pyocyanin, elastase, and rhamnolipid virulence factors. Moreover, upregulation of the CsrA gene established oxidative stress and hindered homeostasis of proteins underlying the mechanism of QSI (Kong et al., 2021). Diazeniumdiolate and leudiazen signal antagonism contributed to mangotoxin QSI in *P. syringae* pv. *syringae in tomato* (Mas-Rosello et al., 2024). Moreover, *P. syringae* pv. *actinidiae* causing citrus canker was affirmed for the non-ribosomal peptides’ antagonism. The corresponding genes were confronted to the AHL acylase gene (*pvdQ*), a glucose-6-phosphate dehydrogenase gene (*zwf*), and an *mbtH*-like gene from the endophytic bacterium, *P. synxantha* (Tontou et al., 2016). Gunpowder green tea extracts showed virulence inhibition of *P. syringae* pv. *actinidiae* virulence mechanisms and QSI (Lovato et al., 2019). Similarly, EPSs from *P. syringae* pv. *actinidiae* NZ V-13 were inhibited by the bactericidal compound kasugamycin in kiwi fruit (Ghods et al., 2015) (**Table 1**). Thus, *P. syringae* QS inhibitors are assessed critically for their comprehensive complexity variation patterns, reflecting a decade of research.

The emerging infectious agent *E. amylovora* was characterized by a QQ mechanism involving EaAiiA lactonase degradation of QSI (Ya’ar Bar et al., 2021). *Moringa oleifera* leaf extracts indicated diminished amylovoran synthesis, biofilm formation, and QSI against *E. amylovora* (Fontana et al., 2022). *Streptomyces rubradiris* NBRC 14000 inhibited the biofilm formation, pyocyanin and rhamnolipid production, swimming motility, and virulence against *E. amylovora* (Xiaoyu et al., 2024). *Erwinia carotovora* subsp*. atroseptica* was inhibited by the QS inhibitors derived from *Streptomyces xanthocidicus* KPP01532 (piericidin A and glucopiericidin) as evident from the expression of virulence genes (*pelC, pehA, celV*, and *nip*) (Kang et al., 2016). *E. amylovora* was inhibited by *P. protegens* 59M through the *phlD, pltC, pltB*, and pltC gene expression and showed potent QSI (**Table 1**). Hence, the mechanisms were corroborated by antagonism, siderophore production, IAA, HCN production, and AHL degradation (Mikiciński et al., 2024). The *Bacillus simplex* showed QQ activities by *the aiiA* gene, which encodes zinc-dependent AHL silencing in *E. amylovora* sy69 (Hanano et al., 2014). Mutations in pyoverdine, safracin, and phenazine from *Pseudomonas orientalis* F9 have demonstrated antagonism against *E. amylovora* in apple flowers, *in vitro* (Santos Kron et al., 2020). *Pantoea ananatis* BCA19 depicted potent biocontrol against *E. amylovora* QSI (siderophore, arimid, arylpolyene, and carotenoid-related terpene gene clusters) (Lee et al., 2024) (**Table 1**). Hence, *E. amylovora* QSI needs repurposing research to prevent crop loss, particularly apple and pear plants.

*X. campestris* accounts for a ubiquitous and versatile phytopathogen of global threat. *Burkholderia anthina* strain HN-8, a novel DSF-degrading bacterium, was identified as a biocontrol agent for black rot disease, caused by *Xcc*. The severity of black rot disease in Chinese cabbage and radishes revealed biocontrol activity upon inoculation of the strain HN-8 (Ye et al., 2020). Chumacin-1 and Chumacin-2, produced by *P. aeruginosa* strain CGK-KS-1, demonstrate DSF inhibition activity in *Xanthomonas oryzae* pv. *oryzae (*Kanugala et al., 2019*).* Infection with *X. campestris* pv. *campestris* in the cabbage plant produces high SA. The turnover of the DSF family QS signal in a pH-dependent way directs the QS system in *X. campestris* pv. *campestris* virulence (Song et al., 2022). *Cupriavidus* sp. reduced black rot caused by *Xcc*. A novel DSF-degrading strain, HN-2, from contaminated soil decreased the severity and proved to be a potent biocontrol agent. Thus, DSF-dependent bacterial infections underlying the biochemical basis were unraveled (Ye et al., 2019a). *Acinetobacter lactucae* QL-1 attenuated *Xcc* virulence through QQ enzymes and revealed the promising potential of plant protection against DSF-dependent pathogens (Ye et al., 2019b). *M. oleifera* Lam leaf extracts depicted QSI against *Xcc* by inhibiting swarming motility and biofilm formation (Fontana et al., 2021). Similarly, *M. oleifera* leaf extracts indicated flavonoids and phenols, revealing effective QSI through biofilm inhibition in *Xcc* (Fontana et al., 2023). Thymol-loaded chitosan nanoparticles (TCNPs) suppressed the growth of biofilm formation and QSI through EPSs and xanthomonadin production against *Xcc* (Sreelatha et al., 2022) (**Table 1**). Hence, the QSI profiles against *Xcc* were critically compiled for intricate properties.

## 8 Challenges and limitations

The incorporation of technology in field applications requires advanced genome editing protocols. Cost-effectiveness and environmental-friendliness, envisaging authentic and reproducible results, are necessitated. Real-world lab-to-field applications and case studies are necessary for accurate outcomes in plant health management. Stringent regulatory and global laws are necessary for ensuring food safety and security. CRISPR/Cas9 technology has been regarded as a cost-effective protocol and robust strategy. Further selectivity and random off-target mutations, as well as homologous recombination, necessitate the implementation of resistance gene knock-out protocols to ensure precision and sensitivity (Wang et al., 2022). CRISPR technology cannot be applied for large-scale field trials and the effectiveness of increased resistance combating phytopathogenic bacteria (Ijaz et al., 2023). Furthermore, ethical issues related to CRISPR/Cas9 technology require global impact and surveillance in sustainable agricultural practices. The flexible regulations are implicated in countries such as the USA, Canada, Argentina, and Australia. Nevertheless, the European Union (EU) and developing nations, such as India, are still a long way from achieving strategic management (Tyagi et al., 2021). Plant–pathogen interactions have been reported to employ CRISPR technology for genome editing in phytopathogenic bacteria (Gosavi et al., 2020). A high level of resistance to the citrus canker disease was conferred by genome editing the promoter region of the S gene CsLOB1 in citrus, which is the target of TALE from *X. citri* pv. *citri* (Peng et al., 2017). DMR6 is essential for *A. thaliana*’s resistance to downy mildew. CRISPR/SpCas9 knocked out homolog SIDMR6-1 in tomatoes, rendering resistance to a variety of bacterial diseases, such as *P. syringae, P. capsica*, and *Xanthomonas* spp. (Thomazella et al., 2016). Additionally, the CRISPR/SpCas9-generated SlJAZ2Δjas tomato germplasm offered resistance to *P. syringae* pv. tomato DC3000 without changing its defense response against the necrotrophic fungal disease *Botrytis cinerea* (Ortigosa et al., 2019). However, CRISPR technology has not been reported to be fruitful against ubiquitous phytopathogens. Thus, limitations are summarized, which need to be addressed in the future.

## 9 Conclusion and future directions

QS and CRISPR systems in plant protection against selected bacterial pathogens, viz, *P. syringae, E. amylovora*, and *X. campestris*, were critically revisited for periodic updates. CRISPR engineering, QS-CRISPR interplay, and limitations in sustainable agricultural practices are outlined for further research. The QS systems in phytopathogenic bacteria are categorized into AHLs, DSFs, and the second messenger cyclic di-GMP. The two-component systems in *P. syringae* consist of RhpRS, GacAS, CvsRS, and AauRS. Moreover, the pathogen employsT3SS s like CorRS TCS, GacAS TCS, LOV-HK TCS, RhpRS TCS, CvsRS TCS, AauRS TCS, and AhlI–AhlR QS systems. *E. amylovora* revealed a T3SS, the EPS amylovoran, biofilm formation, and motility. Complex interaction depicts bis-(3′-5′)-cyclic di-GMP (c-di-GMP), lipopolysaccharide biosynthetic gene clusters, and *hrp* genes confronting virulence. Further TA systems like CbtA/CbeA, ParE/RHH, and Doc/PhD establish severity in plant–microbe interactions. The most frequently reported QS system in *X. campestris* belongs to DSF signals, followed by rpfABFCHG genes. Furthermore, phosphorelay processes based on cyclic di-GMP levels provide interaction between Rpf/DSF QS systems. Additionally, the salicylic acid pathway, sucrose, and glucose levels raise RpfB activity, which in turn raises DSF signals. The interaction between QS and CRISPR may make it more challenging to regulate plant health effectively and ensure sustainable agriculture in the future. CRISPR/Cas9 technology might be used to create climate-resilient farming methods, plant protection, and plant growth enhancement. Furthermore, QS inhibitors concerned with *P. syringae, E. amylovora*, and *X. campestris* were assessed for variability profiles. The future prospective research relies largely upon economic and environmental sustainability. Furthermore, in-depth mechanisms involving plant immunity pathways like salicylic acid– jasmonic acid cross-talk can provoke metabolomics and interactomes involving QSSMs. Elicitors and receptors that contribute to plant immunity, such as systemic acquired resistance (SAR) and induced systemic resistance (ISR), recognize molecular patterns in response to these stimuli, and have been a classical plant mechanism for furthering critical plant growth outcomes (Abdul Malik et al., 2020). Moreover, QSSMs in augmenting phytopathogens inter-kingdom signaling interference are foreseen as the latest realm of research (Joshi et al., 2021). Thus, plant patterns and basic attributes for interaction–interplay in plant immunity are necessitated. Furthermore, DSF-associated signals need to be revisited for deciphering either positive or negative interactions in plant–microbe interaction patterns (Li et al., 2024). QS systems involving SA–JA–ET signaling systems are necessitated for QSSMs and pathogenicity of phytopathogens in the near future (Alagarasan et al., 2017). CRISPR/Cas gene editing offers potential for disease-resistant plants by employing plant immunity mechanisms, such as PTI and/or ETI (Tyagi et al., 2021). Moreover, the technology is also being applied to engineer crop plants resistant to multiple plant pathogens (Zaynab et al., 2020). Harnessing pan-genomes of crop plants and their allelomorphs will aid in the development of climate-resilient and disease-resistant plants with augmented immunity (Kim et al., 2021). CRISPR interference (CRISPRi) of plant pathogens can provide escalated benefits for plants in the future (Arora, 2024). Furthermore, CRISPR technology holds promise for plant defense, breeding, and metabolic engineering (Joshi et al., 2021). Hence, CRISPR editing in the future will enable food safety and security globally. Moreover, microbiome engineering, enhanced plant growth, and disease resistance properties can minimize the impending danger of plant pathogens. Phyto-microbiome engineering through CRISPR/Cas gene editing is expected to improve QSSM-mediated plant immunity, highlighting the interactive roles between plants and microbes (Chaudhary et al., 2021). The future research thrust comprises systems biology and metabolic engineering of signal molecules for dissecting plant–microbe interaction dynamics (Kumar et al., 2016). QQ mechanisms and CRISPR technology stress the need to effectively combat plant pathogens to achieve plant immunity (Sharma et al., 2023).

Genome editing using CRISPR/Cas technology could revolutionize the usage of genetically modified organisms in plant protection globally. Nevertheless, crop improvement and plant performance that abate biotic and abiotic stresses will necessitate explicit research (Wang et al., 2022). Disease-resistant plants with high yields can be combined with CRISPR/Cas9 technology, next-generation sequencing, and multi-omics protocols (Ahmad et al., 2020). The specificity and sensitivity need to be unraveled for increased disease resistance and minimizing off-target mutations (Ijaz et al., 2023). Potential plant breeding can involve CRISPR/Cas9-mediated genome engineering and successive field trials (Sharma et al., 2023). Metabolic engineering using CRISPR technology can aid in disease-resistant and improved varieties (Tyagi et al., 2021). The CRISPR/Cas system and its derivatives provide a novel approach to exploring the complex realm of plant–pathogen interactions (Gosavi et al., 2020). CRISPR/Cas9 technology offers molecular biologists, geneticists, and plant virologists the opportunity to develop crops with increased yields, disease resistance, and plant health (Ahmad et al., 2020). Thus, QS-CRISPR systems in improved plant health management and sustainable agriculture are recapitulated for further prospects.
